# Prevalence and Predictors of Occlusive Myocardial Infarction in Patients Presenting With Non-ST-Elevation Acute Coronary Syndrome in Duhok, Iraq: A Cross-Sectional Study

**DOI:** 10.7759/cureus.65299

**Published:** 2024-07-24

**Authors:** Ameen M Mohammad, Malavan M Ali

**Affiliations:** 1 Internal Medicine, University of Duhok, Duhok, IRQ; 2 Cardiology, Duhok Heart Center, Duhok, IRQ; 3 College of Medicine, University of Zakho, Zakho, IRQ

**Keywords:** acute coronary syndrome, kurdistan region of iraq, occlusive myocardial infarction, total coronary occlusion, non-st elevation myocardial infarction

## Abstract

Background and aim

Myocardial infarction is a major global health issue and the leading cause of death. Non-ST segment elevation acute coronary syndrome (NSTE-ACS) could behave like ST-segment elevation ACS in terms of causing total or near total occlusion of the coronary artery and leading to occlusive myocardial infarction (OMI). This study aims to assess OMI prevalence and associated factors in NSTE-ACS patients in Duhok, Iraq, to improve diagnosis and treatment outcomes.

Materials and methods

This prospective cross-sectional study, conducted at Azadi Heart Center and Zakho Teaching Hospital from March 2023 to March 2024, included 189 NSTE-ACS patients undergoing coronary angiography. Data collection encompassed demographics, clinical profiles, electrocardiographic (ECG) patterns, cardiac biomarkers, and angiographic outcomes. Patients were categorized into those with and without occlusive myocardial infarction (OMI).

Results

A total of 189 NSTE-ACS patients with a mean age of 58.65 (±10.38 SD) years were enrolled in the study. The overall OMI rate was 29.63%. OMI patients were older and had a higher prevalence of hypertension, dyslipidemia, and a family history of ischemic heart disease (IHD). Significant ECG changes associated with OMI included biphasic T-wave inversion and ST depression in specific leads. Marked elevation in troponin levels was also noted in OMI patients. The left anterior descending (LAD) artery was the most common culprit artery.

Conclusions

About one-quarter of our study cohort exhibited OMI. The condition was linked to clinical, ECG, and elevated troponin levels. The study underscores the importance of promptly recognizing occlusive myocardial infarction (OMI) in NSTE-ACS patients for better outcomes. Regular audits are imperative to augment awareness among healthcare professionals at cardiac centers regarding updated protocols and guidelines.

## Introduction

Myocardial infarction remains a formidable global health challenge and the leading cause of mortality worldwide [1]. It represents the most severe clinical manifestation of coronary artery disease [2]. Traditionally, myocardial infarctions are classified based on electrocardiographic (ECG) presentations into ST-elevation myocardial infarction (STEMI) and non-ST elevation myocardial infarction (NSTEMI). STEMI is characterized by a complete coronary artery occlusion, resulting in ST-segment elevation on the ECG, whereas NSTEMI typically indicates partial obstruction without ST elevation [3].

The STEMI/NSTEMI paradigm has long guided emergency cardiology practice. However, recent findings suggest that a significant proportion of non-ST segment elevation acute coronary syndrome (NSTE-ACS) cases may involve a complete or near-complete coronary artery occlusion, categorizing them as occlusive myocardial infarction (OMI) despite the absence of classic ST-segment elevation. This discrepancy can arise from factors such as collateral circulation, the anatomical location of the infarction, and pre-existing conditions that alter ECG presentation [4]. A meta-analysis by Khan et al. found that an average of 25.5% of NSTE-ACS cases were identified as OMI within 24 hours of presentation [5]. Furthermore, the TRITON-TIMI-38 trial analysis indicated that 26.2% of NSTE-ACS patients exhibited completely occluded culprit vessels at angiography [6]. Recognizing OMI in NSTE-ACS patients is crucial due to its significant clinical implications. These patients face a higher risk of adverse outcomes, including increased mortality rates, compared to those with non-occlusive NSTE-ACS. Therefore, prompt and accurate diagnosis is essential for guiding appropriate therapeutic interventions [7].

Understanding the prevalence and characteristics of OMI in NSTE-ACS patients is essential for refining diagnostic criteria and treatment protocols. However, there is currently no data on OMI in NSTE-ACS patients in Iraq. Therefore, our study aims to assess OMI's prevalence and associated factors in NSTE-ACS patients in Duhok, Iraq. This will help enhance physician awareness about this significant paradigm shift in myocardial infarction classification and management, and improve targeted management strategies to significantly enhance outcomes for this high-risk patient cohort.

## Materials and methods

Study design and population

This prospective cross-sectional study was carried out at Azadi Heart Center in Duhok and Zakho Teaching Hospital-Cardiac Unit in the Kurdistan Region of Iraq. The data collection took place between March 2023 and March 2024. A total of 189 patients diagnosed with non-ST-elevation acute coronary syndrome (NSTE-ACS) undergoing coronary angiography within 24 hours up to 21 days of presentation, with or without coronary intervention, were included in the study.

Measurement and parameters

The data collection process focused on four distinct areas for each patient. The first two sections encompassed demographic characteristics, including age and gender, as well as the clinical profile, which included the patient's history of ischemic heart disease (IHD), hypertension, diabetes, dyslipidemia, family history, and smoking status. The subsequent section comprised investigation results, encompassing electrocardiography and cardiac biomarkers. Finally, meticulous recording of angiographic profiles and clinical outcomes was conducted. Interpretation of angiographic analysis and electrocardiographic characteristics was carried out by senior cardiologists and interventionists in the cardiac catheterization laboratories. To achieve the study objectives, patients were categorized into two groups: those with the NSTE-ACS and concomitant occlusive myocardial infarction (OMI), and those with the NSTE-ACS without OMI. Subsequently, their angiographic data were compared, along with the correlation of the patients' clinicodemographic profile and ECG characteristics with the angiographic findings.

Several ECG patterns were considered. These patterns include but are not limited to, the elevation of any degree in two contiguous inferior leads with any amount of ST depression in aVL, which raises suspicion for inferior OMI. Presumed new right bundle branch block (RBBB) and left anterior fascicular block (LAFB) are highly suggestive of proximal left anterior descending (LAD) occlusion. Additionally, small "hyperacute" T-waves, maximal ST depression in leads V1-4 without progression to V5-6, are deemed indicative of posterior OMI, while diffuse ST depression with ST elevation in aVR warrants attention.

Occlusive myocardial infarction (OMI) serves as the anatomic and pathophysiologic substrate of ST-segment elevation myocardial infarction (STEMI), although not all instances of OMI manifest as STEMI. So, the OMI is defined, here as the presence of a lesion with 100% stenosis or thrombolysis in myocardial infarction (TIMI) flow grade 0 to 1 in one or more major coronary vessels, while a non-occluded or patent coronary artery is defined as TIMI flow grade 2 or 3, as observed through invasive coronary angiography.

Inclusion and exclusion criteria

The exclusion criteria encompassed patients who met the ECG standards defined by the fourth universal definition of STEMI, those in cardiogenic shock, cases where angiograms indicated coronary chronic total occlusion exclusively, and individuals lacking essential data such as initial ECG or serum troponin levels or who did not provide informed consent. Inclusion criteria comprised adults aged 18 years and older of both genders presenting with NSTE-ACS who underwent coronary angiography or angioplasty. 

Ethical consideration

The Ethics and Scientific Committee of the Higher Council of Medical Specialties in the Kurdistan Region of Iraq formally approved the conduct of this study on 17th March 2022 with reference number (777). Informed consent was obtained from all patients enrolled in the study. The study was carried out in compliance with the Ethical Standards of the Helsinki Declaration for Medical Research Involving Human Subjects.

Statistical analysis

Statistical analysis was conducted using Microsoft Excel (Microsoft Corporation, Redmond, Washington, United States) and GraphPad Prism version 8 (Dotmatics, California, USA). Initially, the data underwent cleaning and coding in Microsoft Excel, followed by transfer to GraphPad Prism for additional analysis. Categorical data were expressed as numbers and percentages. Continuous data were analyzed using paired t-tests. The association between clinicodemographic profiles and variable outcomes of NSTE-ACS with or without OMI was assessed using the Chi-square test (Fisher’s exact test). A p-value <0.05 is considered statistically significant.

## Results

Clinicodemographic characteristics

Table 1 presents the clinicodemographic characteristics of the sample population. The patients had a mean age of 58.65 years (±10.38 SD). Of the 189 patients, approximately three-fifths were female, and two-fifths were male. About 31.22% had a history of ischemic heart disease (IHD), and 42.86% had a positive family history of coronary artery disease. Additionally, 57.67% of the participants had hypertension, and 41.79% had diabetes. Approximately one-third were smokers, and 37.57% had dyslipidemias.

**Table 1:** Clinicodemographic characteristics of patients (n=189).

Variables	n (%)
Age (mean±SD)	58.65±10.38
Gender	
Male	76 (40.21)
Female	113 (59.79)
Ischemic heart disease (IHD)	
Yes	59 (31.22)
No	130 (68.78)
Hypertension	
Yes	109 (57.67)
No	80 (42.32)
Smoking	
Yes	73 (38.62)
No	116 (61.38)
Diabetes mellitus	
Yes	79 (41.79)
No	110 (58.21)
Dyslipidemia	
Yes	71 (37.57)
No	118 (62.43)
Family history	
Yes	81 (42.86)
No	108 (57.14)

Angiographic findings

Table 2 illustrates the angiography results for patients who presented with NSTE-ACS. The results were categorized as non-OMI and OMI. Among the patients who underwent coronary angiography, 29.63% had occlusive coronary lesions, and 70.37% had non-occlusive lesions.

**Table 2:** Prevalence of OMI in patients with NSTE-ACS. OMI: occlusive myocardial infarction, NSTE-ACS: non-ST segment elevation acute coronary syndrome.

Variables	n (%)
NSTE-ACS without OMI	133 (70.37)
NSTE-ACS with OMI	56 (29.63)

Table 3 summarizes the angiographic distribution of the culprit artery in NSTE-ACS patients. The left anterior descending (LAD) coronary artery was the most frequently involved in patients with OMI, followed by the right coronary artery (RCA) and the left circumflex artery (LCX). This sequence of artery involvement was also observed in patients without OMI. There was a statistically significant difference in the distribution of OMI, non-OMI, and normal cases across the involved arteries.

**Table 3:** Culprit artery in NSTE-ACS patients with and without OMI. LM: left main; LAD: left anterior descending; LCX: left circumflex; OM: obtuse marginal; RCA: right coronary artery; PDA: posterior descending artery; PLV: posterior left ventricle; OMI: occlusive myocardial infarction, NSTE-ACS: non-ST segment elevation acute coronary syndrome. The p-value is determined using Chi-square.

Epicardial coronary artery	NSTE-ACS with OMI n (%)	NSTE-ACS without OMI n (%)	p-value
LM	2 (2.38)	25 (6.81)	0.0053
LAD	29 (34.52)	114 (31.06)	
Diagonal	3 (3.57)	28 (7.63)	
LCX	19 (22.62)	65 (17.71)	
OM	4 (4.76)	25 (6.81)	
RCA	22 (26.19)	74 (20.16)	
PDA	2 (2.38)	16 (4.36)	
PLV	3 (3.57)	20 (5.45)	

Risk factors associated with OMI and non-OMI in NSTE-ACS patients

The mean age of NSTE-ACS with OMI cases was 61.81 years (±10.06 SD), which was significantly higher than that of patients without OMI, whose mean age was 57.21 years (±10.41 SD), with a p-value of 0.006. OMI was more prevalent among patients with hypertension, dyslipidemia, and positive family history, with p-values of 0.002, 0.005, and 0.01, respectively. Other risk factors did not show significant associations. Notably, patients with OMI exhibited significantly elevated troponin levels compared to those without OMI, with a p-value of 0.005. The detailed risk factors associated with OMI are presented in Table 4.

**Table 4:** Risk factors related to NSTE-ACS with and without OMI. The p-value is determined using Chi-square (Fisher exact test). OMI: occlusive myocardial infarction, NSTE-ACS: non-ST segment elevation acute coronary syndrome.

Characteristics	NSTE-ACS with OMI n (%)	NSTE-ACS without OMI n (%)	p-value
Age (mean±SD)	61.81±10.06	57.21±10.41	0.006
Gender			
Male	20 (35.7)	56 (42.11)	0.51
Female	36 (64.29)	77 (57.89)	
Ischemic heart disease			
Yes	16 (28.57)	43 (32.33)	0.73
No	40 (71.43)	90 (67.67)	
Hypertension			
Yes	42 (75)	67 (50.38)	0.002
No	14 (25)	66 (49.62)	
Smoking			
Yes	21 (37.5)	52 (39.10)	0.87
No	35 (62.5)	81 (60.90)	
Diabetes mellitus			
Yes	26 (46.43)	53 (39.85)	0.42
No	30 (53.57)	80 (60.15)	
Dyslipidemia			
Yes	30 (53.57)	41 (30.83)	0.005
No	26 (46.43)	92 (69.17)	
Family history			
Yes	16 (28.57)	65 (48.87)	0.01
No	40 (71.43)	68 (51.13)	
Troponins (ng/l)	2504.744±8335.38	246.71±651.69	0.005

Characteristic ECG changes in NSTE-ACS patients with and without OMI

In patients with OMI, the most common ECG changes included biphasic T-wave inversion, ST depression in lead I and aVL with minimal ST-elevation in inferior leads, and ST depression in leads V1-V4. Table 5 presents a detailed breakdown of ECG characteristics and their association with NSTE-ACS, both with and without OMI, aiding in the analysis of potential links between ECG characteristics and OMI events.

**Table 5:** The pattern of ECG changes in NSTE-ACS patients with and without OMI. The p-value is determined using a paired t-test. OMI: occlusive myocardial infarction, NSTE-ACS: non-ST segment elevation acute coronary syndrome, RBBB: right bundle branch block, LBBB: left bundle branch block.

ECG characteristics	NSTE-ACS with OMI n (%)	NSTE-ACS without OMI n (%)	Total n (%)	p-value
Normal	8 (14.29)	33 (24.81)	41 (21.69)	0.337
ST depression in lead I and aVL with minimal ST elevation in inferior leads	11 (19.64)	2 (1.5)	13 (6.88)	
ST-elevation in aVR	3 (5.36)	1 (0.75)	4 (2.12)	
ST depression V1-V4	6 (10.71)	1 (0.75)	7 (3.70)	
Hyperacute T-wave	3 (5.36)	2 (1.5)	5 (2.65)	
New RBBB	3 (5.36)	3 (2.26)	6 (3.17)	
Biphasic T-wave inversion	8 (14.29)	6 (4.5)	14 (7.41)	
LBBB with modified Sgarbossa criteria	0 (0)	0 (0)	0 (0.0)	
Poor R wave progression	2 (3.57)	2 (1.5)	4 (2.12)	
Others	12 (21.43)	83 (62.41)	95 (50.26)	

## Discussion

To our knowledge, this is the first study in the country to thoroughly investigate the prevalence and characteristics of OMI and non-OMI in NSTE-ACS patients. The insights gained from our research will serve as a valuable resource for physicians in our region, providing them with critical information on when to maintain a heightened suspicion of OMI in cases of NSTE-ACS. This heightened awareness will directly impact clinical decision-making. By aligning their approaches with our study's findings, physicians can optimize patient outcomes and ensure more effective and timely treatment strategies for those affected by NSTE-ACS with suspected OMI. In this study, among NSTE-ACS patients, 29.63% were NSTE-ACS with occluded coronary arteries (OMI), while 70.37% were NSTE-ACS with non-occluded coronary arteries.

In this study, the mean age of NSTE-ACS patients was 58.65 years (±10.38 SD), closely aligning with the mean age reported in another study on NSTEMI patients in Duhok, Kurdistan Region of Iraq [8]. Although studies indicate that men have a 2.4-fold higher risk of NSTEMI compared to women and that women are less likely to undergo revascularization therapy [9]. Notably, about three-fifths of the patients in the current study were female, indicating a female predominance. This finding contrasts with several other studies conducted at the same center, demonstrating a male predominance [10-12]. Furthermore, traditional risk factors for acute coronary syndrome were commonly found among our patients. This clustering of risk factors is consistent with observations from previous reports conducted in the region [13-15].

The study's outcomes revealed that among our studied sample diagnosed with NSTE-ACS, 29.63% presented with occlusive myocardial infarction (OMI), while 70.37% had non-occlusive coronary arteries. This incidence rate of OMI among NSTE-ACS patients aligns with findings from two studies conducted in Turkey and Nepal [4,16]. Similarly, research by Pendell Meyers in the USA indicated that approximately 25%-30% of NSTEMI patients experience acute total occlusion upon diagnosis [17]. However, a study conducted in Pakistan reported a higher occlusion rate of 39% in NSTEMI cases [18]. The varying incidence rates of OMI in NSTE-ACS patients across these studies may be attributed to factors such as delayed patient presentation to the emergency department, misdiagnosis with STEMI and STEMI equivalent cases, procedural delays in performing coronary angiography, and subsequent recanalization of occluded arteries.

The analysis of angiographic data revealed that the left anterior descending artery (LAD) was the most frequently involved vessel in NSTE-ACS patients with OMI in our report, followed by the right coronary artery (RCA) and then the left circumflex artery (LCX). A similar pattern of artery involvement was reported in a study conducted in Saudi Arabia among NSTE-ACS patients [19]. However, our findings differ from those of a larger study by Terlecki et al., which identified the LCX as the most frequent culprit vessel in NSTE-ACS with OMI [20]. This discrepancy may be attributed to differences in sample sizes, patient selection criteria, and sometimes the delayed presentation of STEMI patients to emergency departments, leading to resolved ST-segment elevation on ECG traces and subsequent treatment as NSTEMI. Therefore, timely diagnosis and early referral of ACS cases to capable heart centers and tertiary hospitals are essential for better categorizations and tailored management. 

The major risk factors for NSTE-ACS include smoking, hypertension, diabetes, dyslipidemia, older age, and a family history of ischemic heart disease [8]. In the current study, advancing age, hypertension, dyslipidemia, and positive family history were significantly associated with NSTE-ACS complicated by OMI. However, diabetes and smoking did not show statistical significance. Similar findings were reported in another study, which identified increasing age and family history as significant factors for total coronary artery occlusion in NSTE-ACS cases [16].

Notably, patients with occlusive coronary arteries exhibited markedly elevated troponin levels in this study, a finding also observed in a Saudi Arabian study, which found significant troponin elevation in patients with significant coronary artery disease [21]. The most common ECG patterns associated with totally occluded coronary arteries in our study were normal ECG, ST depression in leads I and AVL with minimal ST-elevation in inferior leads, and biphasic T-wave inversions. Similar ECG findings were reported in an Egyptian study [22]. However, larger cohorts are needed to establish statistically significant correlations. Furthermore, previous reports have demonstrated that occlusion of the LAD in conjunction with risk factors, dynamic ECG changes, and elevated troponin levels are associated with hemodynamic instability and unfavorable clinical outcomes in ACS cases [23]. Moreover, strain echocardiography and inflammatory markers, such as high-sensitive C-reactive protein, fibrinogen to albumin ratio, and neutrophil to lymphocyte ratio may aid in diagnosing acute vessel closure in cases of ACS and are linked to adverse prognostic outcomes [24,25].

Strengths and limitations

Our study had limitations. Firstly, the time-limited study population made it challenging to achieve statistical significance for differences in some risk factors between NSTE-ACS patients with and without OMI. Secondly, delays in performing angiographic evaluations may have affected the identification and classification of occlusive coronary arteries, potentially leading to an underestimation of OMI prevalence. Additionally, the lack of a follow-up study prevented us from assessing long-term clinical outcomes and the effects of revascularization, highlighting the need for future studies with long-term follow-up. Despite these limitations, our study is notably strengthened as it represents the first research in the country to assess the prevalence and associated factors of occlusive coronary arteries in patients presenting with NSTE-ACS in more than a single center in Iraq. 

Recommendations

This effort provides valuable baseline data and highlights critical clinical data that will aid physicians and junior house officers in the early identification and suspicion of OMI in patients presenting with NSTE-ACS in our country, thereby improving outcomes for high-risk patients. The Azadi and Zakho Heart Centers should consider establishing periodic audits to enhance centers' healthcare workers' awareness and knowledge about new protocols and information.

Current guidelines recommend an immediate invasive strategy for coronary revascularization within two hours for NSTE-ACS patients with refractory angina, hemodynamic or electrical instability, and an early invasive approach within 24 hours for patients with elevated risk scores [26]. However, meta-analysis and observational studies indicate a high percentage of OMI cases identified within 24-hour angiograms, suggesting that these clinical criteria and guidelines may not reliably exclude or diagnose NSTEMI with suspected OMI [27,28]. Therefore, we recommend that cardiac centers in Iraq work to reduce the time to intervention for NSTE-ACS patients as much as possible because at least one-third of NSTE-ACS cases in the country behave as STE-ACS.

To address the limitations of the present study, we recommend involving more centers from Iraq in such studies. These studies should incorporate timely angiographic assessments and include long-term follow-up to better understand the clinical outcomes and effectiveness of revascularization strategies in patients with NSTE-ACS and OMI. Such comprehensive research will be instrumental in enhancing patient care and optimizing treatment protocols.

## Conclusions

In our study, we found that approximately one-third of NSTE-ACS patients presented with totally occluded coronary arteries. The risk factors predicting OMI among our study sample included advanced age, hypertension, dyslipidemia, and a positive family history. Patients with OMI exhibited significantly higher troponin levels compared to those without OMI. The ECG findings associated with OMI patients were ST depression in specific leads, and biphasic T-wave inversion. Notably, the left anterior descending (LAD) artery, a major supplier of blood to the myocardium, was frequently identified as the culprit vessel in OMI cases.
